# Case Report: Living donor liver transplantation for the treatment of recurrent pediatric acute liver failure with neuroblastoma amplified sequence gene mutation: a literature review

**DOI:** 10.3389/fped.2025.1527759

**Published:** 2025-04-30

**Authors:** Shengying He, Yi Zhao, Zhu jin, Xinrong Xia, Chengyan Tang, Yuan Gong, Lu Huang, Qing Du, Daiwei Zhu, Wankang Zhou, Yuanmei Liu, Zebing Zheng

**Affiliations:** ^1^ Department of Pediatric Surgery, Children’s Hospital of Guizhou, Affiliated Hospital of Zunyi Medical University, Zunyi, Guizhou, China; ^2^ Guizhou Children’s Hospital, Affiliated Hospital of Zunyi Medical University Zunyi, Zunyi, Guizhou, China

**Keywords:** neuroblastoma amplified sequence, acute liver failure, living donor liver transplantation, pediatric liver, gene mutation

## Abstract

**Background:**

Biallelic mutations in the neuroblastoma amplified sequence (*NBAS*) gene can cause recurrent acute liver failure (RALF) and multi-systemic disease.

**Case presentation:**

Herein, we report a 3-year-old Chinese boy with RALF due to a novel heterozygote mutation c.3596G>A(p.C1199Y)/c.1028G>A(p.S343N) in the *NBAS* gene, identified by whole-exome sequencing. The missense mutation c.3596G>A(p.C1199Y) was inherited from his father, and c.1028G>A(p.S343N) was inherited from his mother. He had suffered six acute liver crises triggered by fever. He eventually underwent living donor liver transplantation (LDLT) at 44 months, with his father donating the left lateral lobe liver, and is now healthy with no recurrence of ALF.

**Conclusion:**

We describe a novel pathogenic mutation in the *NBAS* gene of a patient with RALF and report that LDLT is a safe and efficient treatment for RALF caused by the *NBAS* gene mutation.

## Introduction

Pediatric acute liver failure (ALF) is a life-threatening condition with different phenotypes according to age and underlying etiology, which includes infection, inborn errors of metabolism, drug poisoning, abnormal perfusion, and autoimmune diseases (1). Although etiology is an important factor in determining prognosis and treatment, the etiology of up to 50% of children with ALF remains unknown (1). In 2015, whole-exome sequencing (WES) identified neuroblastoma amplified sequence (*NBAS*) gene mutations as a novel etiology of recurrent ALF (RALF) (2). The *NBAS* gene is located on chromosome 2p24.3 and has 52 exons encoding a protein called NBAS, which contains 2371 amino acids and is involved in intracellular vesicular trafficking (3). The NBAS protein is broadly expressed in various organs, including the liver, heart, skeletal muscle, and leukocytes. Many studies reported that *NBAS* gene mutations were associated with infantile failure syndrome 2 (ILFS2), a fever-induced multi-systemic syndrome known as short stature with optic atrophy and Pelger-Huët anomaly (SOPH) syndrome, and immunodeficiency (4, 5). Here, we report the possible first case of RALF caused by a novel compound heterozygous mutation in the *NBAS* gene that underwent living donor liver transplantation (LDLT) in China. We have described in detail the clinical features, genetic variants, and longer-term outcomes and discussed the similarities and differences with cases reported in the literature.

## Case presentation

A 3-year-old boy with a history of RALF was admitted to our hospital due to a high fever (39.0℃) for six hours. He had no symptoms of cough, runny nose, headache, dizziness, coma, shortness of breath, chest tightness, dyspnea, or incontinence. This was the sixth time that he had RALF, which began at 11 months and recurred at 14, 26, 31, and 39 months, respectively. Physical examination (PE) revealed mild malaise and drowsiness, normal intelligence and behavior, hemorrhage-free skin, slight tenderness in the upper abdomen, mild percussion in the liver area, and no visual or hearing impairment, liver enlargement, or rebound pain. A neurological examination showed normal muscle tension and strength and a normal Babinski sign. Laboratory examination of the liver function tests revealed the following results: alanine transaminase (ALT) 4,682 U/L, aspartate transaminase (AST) 8,352 U/L, total bilirubin (TB) 20.2 μmol/L, direct bilirubin (DB) 10.4 μmol/L, alkaline phosphatase 343 U/L, γ-glutamyl-transferase (γ-GGT) 35 U/L, and total bile acid 387.69 μmol/L. Coagulation parameters included a prothrombin time (PT) of 16.6 s, an international normalized ratio (INR) of 1.45, thrombin time (TT) of 17.4 s, and blood ammonia of 85 μmol/L. Test for Epstein–Barr virus, cytomegalovirus, and other hepatitis viruses were all negative. Autoimmune hepatitis-related autoantibodies were also negative. An ultrasound showed no abnormalities in the heart, liver, or spleen. Brain magnetic resonance imaging was normal.

Immunologic evaluation of the patient showed that serum levels of immunoglobulin (Ig) A (0.62 g/L) and IgG (6.3 g/L) were slightly decreased, and IgE (224 IU/ml) was slightly increased. The serum level of complement C3 was 0.70 g/L. The serum immune cell count showed the absolute values of total immune cells, B lymphocytes, and T helper cells as 4,108 μl, 732 μl, and 1,635 μl, respectively, which were considerably higher. However, the absolute value of natural killer (NK) cells was normal.

Genomic DNA extracted from peripheral blood cells of the patient was subjected to WES. The data showed that the patient had a novel compound heterozygote mutation of c.3596G>A (p.C1199Y) and c.1028G>A (p.S343N) in the *NBAS* gene. Parental genotyping confirmed the mutation c.3596G>A (p.C1199Y) in the father and the c.1028G>A (p.S343N) mutation in the mother. The c.3596G>A (p.C1199Y) mutation has been previously reported in many Chinese patients with ALF, which is characterized by a highly conserved amino acid residue that affects the structure and function of the NBAS protein and is categorized as pathogenic (6). However, the c.1028G>A (p.S343N) is a novel mutation that has not been reported in the referencing or ClinVar databases so far. According to the MutationTaster database, all the mutations were predicted to be pathogenic. The PolyPhen database predicted that each parental variant would have a deleterious effect on the NBAS protein function.

**Figure 1:**
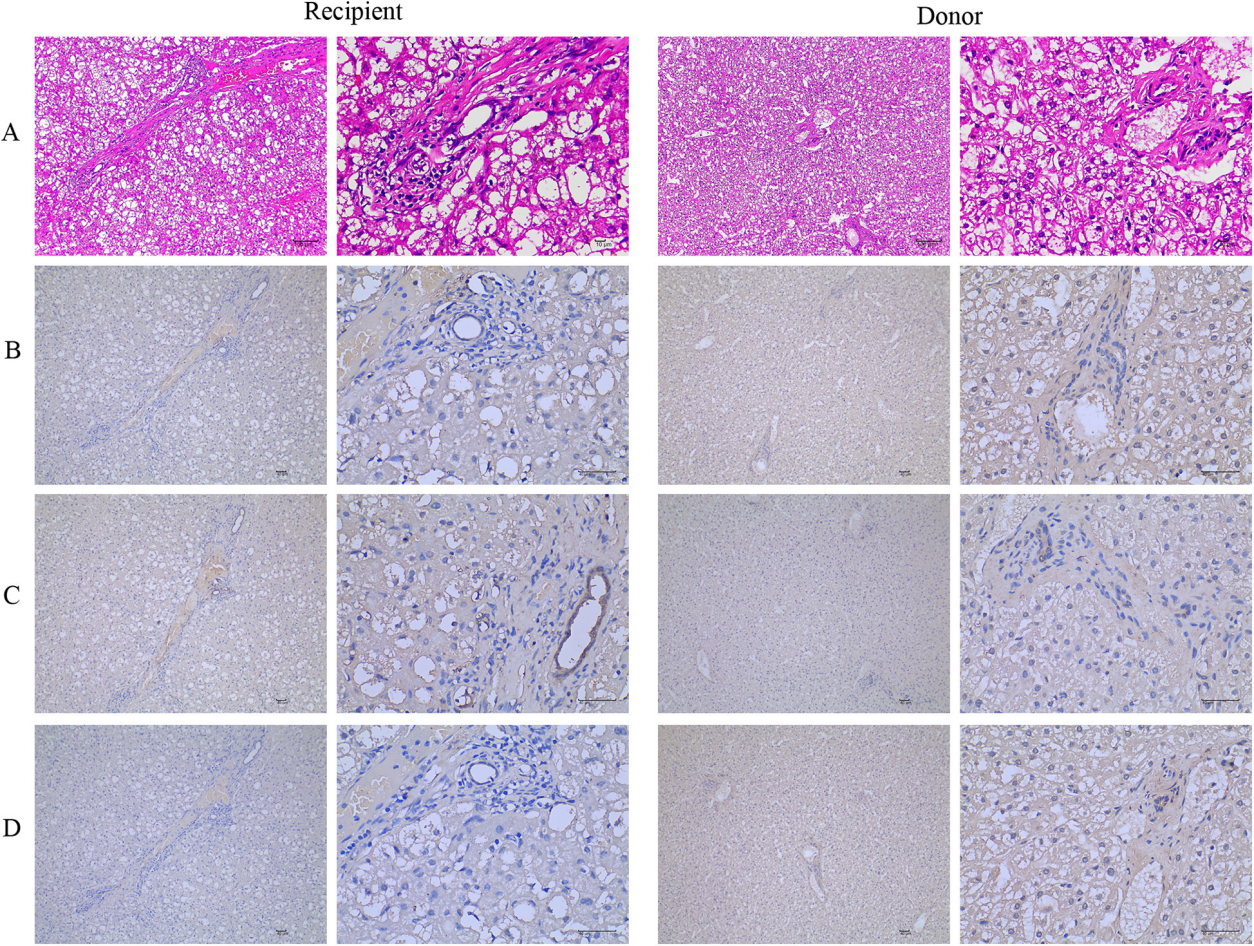
The *NBAS* mutation causes microvesicular steatosis and fibrosis. **(A)** Hematoxylin-eosin (H&E) stained liver biopsy shows hepatocytes with vacuolization, many lymphocytes and neutrophils infiltrated, and mild fibrosis in the portal area on routine compared with his father's hepatocytes. **(B)** The NBAS immunohistochemistry stained in the liver showed that the level of NBAS was significantly decreased in the patient compared with his father's hepatocytes. **(C)** Positive cytokeratin 7 stained ductular proliferations. **(D)** The perilipin 2 immunohistochemistry stain shows mild microvesicular steatosis. NBAS, neuroblastoma amplified sequence.

The patient and his father underwent percutaneous core needle liver biopsy. The patient's liver biopsy was performed after his sixth liver crisis, when he was three years and six months old. Transaminases were normal (ALT/AST = 36/34), until five days prior to biopsy. On routine hematoxylin-eosin (H&E) staining, the patient's liver architecture showed hepatocytes preserved with vacuolization, infiltration of lymphocytes and neutrophils, and mild fibrosis in the portal area. These findings were compared with his father's hepatocytes (Figure 1A). The NBAS immunohistochemistry staining in the liver showed that the level of NBAS was significantly lower in the patient than in the his father's liver (Figure 1B). Cytokeratin 7 staining showed positive results that highlighted ductular proliferations, indicating the sign of cholestasis (Figure 1C). The perilipin 2 immunohistochemistry staining showed that the patient's liver had mild microvesicular steatosis compared to his father's liver (Figure 1D).

## Results

After a thorough discussion with his family, the patient's father, aged 31 years with a height and body weight of 165.0 cm and 63.0 kg, respectively, wanted to donate a portion of his liver for LDLT. The donor's blood test results indicated the liver function, coagulopathy, and immunologic evaluation were normal. The donor's liver biopsy showed normal results.

Pretransplant liver volumetry as measured using IQQA-3D liver (EDDA Technology, lnc., Shanghai, China) showed that the donor's whole liver volume was 1,728 ml and the left lateral segment volume was 340 ml. The donor underwent left lateral segmentectomy for LDLT. The operation time was 2 h and 20 min, and the blood loss was 120 ml. No transfusions were administered during the operation. Finally, the donor's left lateral segment graft weight was 310 g. He was discharged from the hospital on postoperative day (POD) 8.

The recipient's height and body weight were 100.0 cm and 15.0 kg, respectively, and the standard liver volume was 480 ml. The recipient underwent LDLT using the donor's left lateral segment graft. First, the hepatic outflow of graft implantation was anastomosed to the open end of the recipient's hepatic vein, which was extended with an incision at the inferior vena cava, and then the portal vein was reconstructed. Under a microscope, the graft hepatic artery was anastomosed to the recipient's left hepatic artery. The biliary reconstruction was a duct-to-duct biliary anastomosis without T-tube insertion. The operative time was 6 h and 30 min, and the blood loss was 150 ml. There were no blood transfusions during the operation. The cold and warm ischemic times of the graft were 20 and 35 min, respectively. The graft to recipient weight ratio (GRWR) calculated by the weight of the patient was 2.1%.

The immunosuppressive regimen consisted of tacrolimus, mycophenolate mofetil, and methyl­prednisolone. The liver function measured immediately after operation showed the levels of serum transaminase were very high at POD 1 (ALT 1,269 U/L and AST 1,473 U/L), but gradually returned to normal at POD 19. The level of GGT rose to 112 U/L at POD 1 and returned to normal at POD 7. The coagulation parameters, including PT, APTT, and TT, were abnormal immediately after the operation but gradually returned to normal at POD 5. The abdominal drainage tube was pulled out at POD 6. However, the ascites appeared again at POD 10, and the ascites improved after another abdominal puncture, tube drainage, and infusion of human albumin for three weeks. While monitored using Doppler ultrasound and enhanced computed tomography scans, there were no strictures in the hepatic arteries, portal veins, or hepatic veins,. There were no episodes of rejection or surgical complications during recovery or follow-up. Currently, the patient's liver functions are normal when fever after LDLT.

## Discussion and conclusions

The *NBAS* gene encodes a protein that includes two leucine zipper domains, namely a ribosomal protein S14 motif and a Sec39 domain (1). NBAS protein is thought to be a component of an endoplasmic reticulum (ER) tethering complex that interacts with the t-SNAREs p31, BNIPI, and STX18 at the ER and v-SNARE, which regulate the docking and fusion between target membrane and transport vesicles (7). Because of this, the mechanism of fever-dependent ALF in the *NBAS* mutation is related to altered golgi-ER retrograde transport and ER stress.

We presented a Chinese pediatric RALF case with two mutations, c.3596G>A (p.C1199Y) and c.1028G>A (p.S343N), in the *NBAS* gene. As far as it is understood, the c.3596G>A(p.C1199Y) mutation is located in the strictly conserved Sec39 domain of the *NBAS* gene, which can lead to structural changes of the whole Sec39 region or the entire protein (8). Sec39 was first found to be a component of the Dsl1p complex involved in the reverse transcriptional transport from the golgi apparatus to the ER in yeast as a protein. Further study identified that *NBAS* contains a Sec39 domain, which is a direct homologous sec39 protein (7). In humans, NBAS protein participates in retrograde transport from the golgi apparatus to the ER via the formation of the synaptic fusion protein 18 complex (7). *In silico* analysis predicated on the c.1028G>A(p.S343N) mutation had a deleterious effect on protein function, which was also reported in this study. However, further studies with a larger sample size are needed to confirm our observation.

## Literature review on NBAS mutation with liver transplantation

There are currently 101 patients with *NBAS* mutations described in the literature (including the one patient in our study), with an overall survival rate of 89% (90/101). To date, seven (6.9%, four females and three males; six in the previous literature and one patient from our study) out of 101 patients underwent LT. The median age at the initial RALF was 18.3 months (interquartile range, IQR 9–48), and the median age at the LT was 43.9 months (IQR 15–72). One patient was associated with SOPH syndrome. The average number of ALFs was 3.3 (IQR 2–6). Four patients underwent LDLT operations, and three underwent deceased donor LT (DDLT) operations. All the patients who underwent LT experienced no RALF and were alive at the time of the respective study (Table 1).

**Table 1:** Summary of reports of NBAS mutation requiring liver transplantation.

Case no, (Ref.)	Reference	Age at initial RALF(months)	Age at LT (months)	Sex	No. of ALF	No. of Liver Crises without ALF	Liver Biopsy Results	HE Grade	Operation Type	NBAS Mutations	Associated symptoms	Observation Period(years)	Recurrence of LC after LT	Outcome
1	[Ref. (1)]	48	50	F	3	2	Periportal fibrosis	1–2	LDLT	c.1,549C>T(p.R517C) c.4646T>C(p.L1549P)	SOPH	2	No	Alive
2	[Ref. (2)]	9	60	F	5	10	Mis, Mas, fibrosis, Ductular	4	LDLT	c.3602A>C(p.Gln120pro) c.3602A>C(p.Gln120pro)	None	9	No	Alive
3	[Ref. (3)]	26	72	F	2	2	Mas, Ductular	–	LDLT	c.1628-1629lnsA p.Ser544ValfsTer11 c.1226C>T(p.Ala409Val)	None	3	No	Alive
4	[Ref. (4)]	14	23	M	2	3	Fibrosis	–	DDLT	c.2822G>A(p.Arg941His)/-	None	1	No	Alive
5	[Ref. (5)]	9	15	F	2	0	Portal fibrosis, lobular dissecans Mas	2	DDLT	c.2708T>G(p.Leu903Arg)/-	None	0.5	No	Alive
6	[Ref. (6)]	11	45	M	3	0	Mas, coagulative central necrosis	–	DDLT	c.809G>C(2926del),p.Gly270Ala,Ser976Profs*16/-	None	8	No	Alive
7	[Current study]	11	44	M	6	–	Fibrosis, microvesicular steatosis, cholestasis	4	LDLT	c.3596G>A (p.C1199Y)/c.1028G>A (p.S343N)	None	1	No	Alive

The livers of seven LT patients were analyzed histologically. The most common observations were fibrosis, macrovesicular steatosis, microvesicular steatosis, and coagulative central necrosis. Four of the LT patients had hepatic encephalopathy (HE), ranging from grade 1–4.

## Clinical implications and recommendations

Although the severity of RALF episodes varies, most of the research reported cases were recovered with individualized supportive treatment and close follow-up. The overall survival rate was 89% (90/101) and only 6.9% (7/101) underwent LT. At present, there is a lack of detailed criteria for evaluating RALF LT indicators because of the low rates of LT, as well as their role and use in preventing further attacks and prognosis after LT. Calvo et al. reported that LT was required during one of the fulminant crises, but the final diagnosis was later confirmed by using genetic analysis (9). Geem et al. reported that LT should be considered when a patient develops a cerebral hemorrhage and it becomes difficult to manage with medical treatment (5). Many patients with RALF who present with extremely severe crises, polyvisceral failure, and severely impaired liver function suggest that patients require LT. In our series, the patient presented with six liver crises, and the most serious one was a coma in the intensive care unit (ICU) for 10 days due to HE at 31 months. At the age of three years, we evaluated the degree of liver damage by liver biopsy that showed liver with steatosis, hepatocyte swelling, and H&E staining showed mild periportal fibrosis. The immunohistochemistry showed cholestasis and microvesicular steatosis in the liver tissue. The results of the liver biopsy are consistent with the pathological findings of the *NBAS* mutation that have already been reported. Staufner et al. observed that patients with *NBAS* mutations who were followed up without LT were noted to have ongoing liver fibrosis (3). Suzuki et al. reported a 34-year-old patient with the *NBAS* mutations who had a cessation of acute attacks in early childhood but later developed symptoms of cirrhosis (10). Therefore, we recommend that the *NBAS* mutation with extremely recurrent liver crisis, HE, ongoing hepatic fibrosis, and polyvisceral failure, which is difficult to manage with medical treatment, be treated with LT as soon as possible.

In Europe, about 15%–20% of children with liver disease die every year while waiting for LT (11). At present, LDLT and DDLT are the most commonly used LT methods for the treatment of *NBAS* mutations (5). The emergence of LDLT has greatly eased the tension between supply and demand. Around 50% of the mutated genes come from one of their parents in RALF, caused by the *NBAS* mutation. Therefore, the gene phenotype and appropriate donor liver volume of their parents should be identified before adopting LDLT. A liver biopsy can be performed to determine whether there is fibrosis in the liver and the expression of NBAS protein, to reduce the occurrence of postoperative complications and avoid the recurrence of postoperative liver failure. In our series, the patient's donor was his father, who was evaluated by gene sequencing and liver biopsy before the LT operation. The results indicated that the donor was carrying the c.3596G>A (p.C1199Y) mutation, with normal levels of NBAS protein and without liver fibrosis. At the follow-up after LD-LT, the recipient's liver functions were normal but he had a fever. Many studies reported cases in which the liver remained healthy after transplantation, with no further episodes of ALF (5, 12, 13). With the continuous development of LT, more and more children with ALF may be treated.

In summary, we have reported that a novel compound heterozygote mutation in the *NBAS* gene caused RALF in a Chinese child and the resolution of RALF episodes after LDLT. The patient continues to be healthy during regular post-transplant follow-up. We also put forward the advice of LT for this kind of patient with our current experience of LT for the *NBAS* mutation.

## Data Availability

The original contributions presented in the study are included in the article/Supplementary Material, further inquiries can be directed to the corresponding author.
